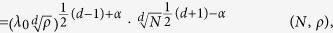# Erratum: Tailoring superradiance to design artificial quantum systems

**DOI:** 10.1038/srep28121

**Published:** 2016-07-07

**Authors:** Paolo Longo, Christoph H. Keitel, Jörg Evers

Scientific Reports 6: Article number: 2362810.1038/srep23628; published online: 03242016; updated: 07072016

This Article contains formatting errors in the labeling of the following Equations,

In Equation 16,


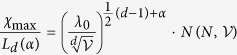


should read:


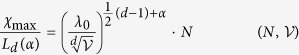


In Equation 17,


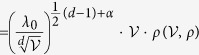


should read:


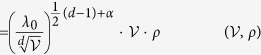


In Equation 18,


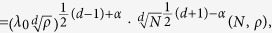


should read: