# Supplementary material for: Tailoring superradiance to design artificial quantum systems
Source: Sci Rep. 2016 Mar 24;6:23628. doi: 10.1038/srep23628 (PMC4806359; doi:10.1038/srep23628)
Supplement: Supplementary Information [file srep23628-s1.pdf]

Tailoring superradiance to design artificial quantum systems
Supplementary information

Paolo Longo, Christoph H. Keitel, and Jörg Evers
Max Planck Institute for Nuclear Physics, Saupfercheckweg 1, 69117 Heidelberg, Germany

In this supplementary information, we provide technical details on how to rewrite the lattice sums into an integral and on how to ultimately perform the integration.

I. $\mathcal{I}_d(\mathbf{k})$

The quantity

$$\mathcal{I}_d(\mathbf{k}) = \sum_{n_1}{}' \cdots \sum_{n_d}{}' V_{a\sqrt{n_1^2+\cdots+n_d^2}} \mathrm{e}^{-\mathrm{i}k_1 a n_1} \ldots \mathrm{e}^{-\mathrm{i}k_d a n_d} \,, \tag{1}$$

where the sums run over all combinations of $\{n_i\}$ except $n_1 = \cdots = n_d = 0$, can be rewritten into an integral

$$\mathcal{I}_d(\mathbf{k}) \to \int \frac{\mathrm{d}^d x}{a^d} V(r,\theta) \prod_{j=1}^{d} \mathrm{e}^{-k_j x_j} \,. \tag{2}$$

For $d = 1, 2$, $V(r,\theta) = f_r$, whereas for $d = 3$, $V(r,\theta) = \sin^2\theta\ f_r$ ($f_r \equiv A_d \exp(\epsilon \mathrm{i} k_0 r)/(k_0 r)^\alpha$). Here, $\mathrm{d}^d x$ signifies the d dimensional infinitesimal volume element and the integration is over all space except for a region with radius a around the origin ($\mathrm{d}^1 x = \mathrm{d}x$, $\mathrm{d}^2 x = r\mathrm{d}r\mathrm{d}\varphi$, $\mathrm{d}^3 x = r^2 \sin\theta \mathrm{d}r\mathrm{d}\varphi\mathrm{d}\theta$).

Explicitly, for $d = 1$,

$$\begin{aligned}\mathcal{I}_1 &= \int_{-\frac{Na}{2}}^{\frac{Na}{2}} \frac{\mathrm{d}x}{a} \Theta(|x| - a) f_{|x|} \mathrm{e}^{-\mathrm{i}kx} \\ &= \int_{a}^{\frac{Na}{2}} \frac{\mathrm{d}x}{a} f_{|x|} 2\cos(kx) \\ &= 2\int_{a}^{\frac{Na}{2}} \frac{\mathrm{d}r}{a} f_r \cos(kr) \,,\end{aligned} \tag{3}$$

where $k = |\mathbf{k}|$ and $\Theta(\cdot)$ signifies the Heaviside step function. For $d = 2$,

$$\begin{aligned}\mathcal{I}_2 &= \int_a^{N_2' a} \int_0^{2\pi} \frac{r\mathrm{d}r\mathrm{d}\varphi}{a^2} f_r \mathrm{e}^{-\mathrm{i}k_1 r\cos\varphi} \mathrm{e}^{-\mathrm{i}k_2 r\sin\varphi} \\ &= \frac{2\pi}{a^2} \int_a^{N_2' a} \mathrm{d}r\ r f_r J_0(kr) \,,\end{aligned} \tag{4}$$

where N_2' is chosen such that the integration area covers N atoms, i. e., $\pi(N_2')^2 = N$. For $d = 3$,

$$\begin{aligned}\mathcal{I}_3 &= \int_a^{N_3' a} \frac{r^2 \mathrm{d}r}{a^3} \int_0^\pi \mathrm{d}\theta \sin\theta \int_0^{2\pi} \mathrm{d}\varphi \underbrace{V(r,\theta)}_{=\sin^2\theta\ f_r} \\ &\quad \times \mathrm{e}^{-\mathrm{i}k_1 r\sin\theta\cos\varphi} \mathrm{e}^{-\mathrm{i}k_2 r\sin\theta\sin\varphi} \mathrm{e}^{-\mathrm{i}k_3 r\cos\theta} \\ &= 2\pi \int_a^{N_3' a} \frac{r^2 \mathrm{d}r}{a^3} f_r \int_0^\pi \mathrm{d}\theta \sin^3\theta \mathrm{e}^{-\mathrm{i}k_3 r\cos\theta} \\ &\quad \times J_0\left(\sin\theta \sqrt{k_1^2 + k_2^2}\ r\right) \,,\end{aligned} \tag{5}$$

where $(4\pi/3)(N_3')^3 = N$, $J_0(\cdot)$ signifies the zeroth-order Bessel function of first kind, and $\mathrm{sinc}(x) = \sin(x)/x$. The integration over θ can be done as follows. Upon defining $I_m \equiv \int_0^\pi \mathrm{d}\theta \sin^m\theta \exp(-\mathrm{i}k_\perp r\cos\theta) J_0(k_\parallel r\sin\theta)$, $k_\perp \equiv k_3$, and $k_\parallel \equiv \sqrt{k_1^2 + k_2^2}$, we have the relation

$$\begin{aligned}I_3 &= I_1 - \int_0^\pi \mathrm{d}\theta \cos^2\theta \sin\theta \mathrm{e}^{-\mathrm{i}k_\perp r\cos\theta} J_0(k_\parallel r\sin\theta) \\ &= \left(1 + \frac{1}{r^2}\frac{\partial^2}{\partial k_\perp^2}\right) I_1 \,.\end{aligned} \tag{6}$$

To simplify the integration needed for I_1, we can choose a coordinate system in which either $k_\perp = 0$ or $k_\parallel = 0$ (it can be shown that I_1 does not depend on the orientation of $\mathbf{k}$), yielding

$$I_1 = 2\ \mathrm{sinc}\left(\sqrt{k_\perp^2 + k_\parallel^2}\ r\right) \,. \tag{7}$$

Finally, $I_3 = \sin^2\vartheta \cdot 2\mathrm{sinc}(kr) + \mathcal{O}[(kr)^{-2}]$ and therefore

$$\mathcal{I}_3 = \frac{4\pi}{a^3} \sin^2\vartheta \int_a^{N_3' a} \mathrm{d}r\ r^2 f_r \mathrm{sinc}(kr) \,, \tag{8}$$

where ϑ denotes the angle between the eigenstate's wavevector $\mathbf{k}$ and the z axis. Here, we have only taken into account the asymptotic leading order term (with respect to kr). Other terms can be accounted for by means of different coefficients α (see main text). Introducing the abbreviations used in the paper and performing a variable substituion, we finally arrive at the integrals $\mathcal{J}_d(k)$ (Eq. (14) in the main text).

II. $\mathcal{J}_d(k)$

We now proceed with the radial integration

$$\mathcal{J}_d(k) = \int_{k_0 a}^{k_0 a b_d \sqrt[d]{N}} \mathrm{d}\eta\ \mathrm{e}^{\pm\mathrm{i}\eta} g_d(k k_0^{-1}\eta)\ \eta^\beta \,. \tag{9}$$

Note that for $d = 2$ the integration kernel is actually given by $J_0(k k_0^{-1}\eta)$. However, already at the lower integration limit, we can use the asymptotic form $J_0(ka) \approx \sqrt{2/\pi}\cos(ka - \pi/4)/\sqrt{ka}$ since in an extended sample $k_0 a > 1$ and only the wavenumbers k around k_0 are relevant. Furthermore, by an additional substituion of the integration variable, we shift the $\pi/4$ shift to the

argument to the exponential (which we can account for by means of appropriate prefactors), $\eta + \pi/4 \approx \eta$, and the integration limits can also approximately remain unchanged.

The possible combinations in the integrand we need to consider are

$$J_{\rm cc} \equiv \int_{k_0 a}^{k_0 a b_d \sqrt[d]{N}} \mathrm{d}\eta \;\; \cos(\eta)\cos(k k_0^{-1}\eta)\; \eta^\beta \,, \tag{10}$$

$$J_{\rm sc} \equiv \int_{k_0 a}^{k_0 a b_d \sqrt[d]{N}} \mathrm{d}\eta \;\; \sin(\eta)\cos(k k_0^{-1}\eta)\; \eta^\beta \,, \tag{11}$$

$$J_{\rm cs} \equiv \int_{k_0 a}^{k_0 a b_d \sqrt[d]{N}} \mathrm{d}\eta \;\; \cos(\eta)\sin(k k_0^{-1}\eta)\; \eta^\beta \,, \tag{12}$$

$$J_{\rm ss} \equiv \int_{k_0 a}^{k_0 a b_d \sqrt[d]{N}} \mathrm{d}\eta \;\; \sin(\eta)\sin(k k_0^{-1}\eta)\; \eta^\beta \,. \tag{13}$$

A. $J_{\rm cc}$

Utilizing a computer algebra system, we find that

$$\begin{aligned} J_{cc} = \frac{\mathrm{i}^{\beta+1}}{4} \cdot \Bigg[& \left(\frac{r_-}{k_0}\right)^{-1-\beta} (\mathrm{sgn}(r_-))^{-2\beta} \\ & \times \Big(\Gamma(1+\beta, -\mathrm{i}r_- a) \\ & \quad - \Gamma(1+\beta, -\mathrm{i}r_- a N_d') \\ & \quad - (-1)^\beta \Gamma(1+\beta, \mathrm{i}r_- a) \\ & \quad + (-1)^\beta \Gamma(1+\beta, \mathrm{i}r_- a N_d')\Big) \\ + & \left(\frac{r_+}{k_0}\right)^{-1-\beta} \\ & \times \Big(\Gamma(1+\beta, -\mathrm{i}r_+ a) \\ & \quad - \Gamma(1+\beta, -\mathrm{i}r_+ a N_d') \\ & \quad - (-1)^\beta \Gamma(1+\beta, \mathrm{i}r_+ a) \\ & \quad + (-1)^\beta \Gamma(1+\beta, \mathrm{i}r_+ a N_d')\Big)\Bigg] \,, \end{aligned} \tag{14}$$

where $r_\pm \equiv k \pm k_0$ and $\Gamma(a,z) \equiv \int_z^\infty \mathrm{d}t \; t^{a-1} \mathrm{e}^{-t}$ signifies the incomplete Gamma function. The asymptotic form for $N_d' \gg 1$ and $r_- \neq 0$ reads

$$\begin{aligned} J_{cc}(k \neq k_0) \simeq \frac{\mathrm{i}^{\beta+1}}{4} \cdot \Bigg[& \left(\frac{r_-}{k_0}\right)^{-1-\beta} (\mathrm{sgn}(r_-))^{-2\beta} \\ & \times \Big(\Gamma(1+\beta, -\mathrm{i}r_- a) \\ & \quad - (-1)^\beta \Gamma(1+\beta, \mathrm{i}r_- a) \\ & \quad - 2\mathrm{i}(-1)^\beta (\mathrm{i}r_- a N_d')^\beta \sin(r_- a N_d')\Big) \\ + & \left(\frac{r_+}{k_0}\right)^{-1-\beta} \\ & \times \Big(\Gamma(1+\beta, -\mathrm{i}r_+ a) \\ & \quad - (-1)^\beta \Gamma(1+\beta, \mathrm{i}r_+ a) \\ & \quad - 2\mathrm{i}(-1)^\beta (\mathrm{i}r_+ a N_d')^\beta \sin(r_+ a N_d')\Big) \,. \end{aligned} \tag{15}$$

The dominant terms for $N_d' \gg 1$ in this expression are $\propto (N_d')^\beta$ if $\beta > 0$.

For $k \to k_0$ ($N_d' = b_d \sqrt[d]{N} \gg 1$), we arrive at

$$J_{cc}(k \to k_0) \simeq \frac{1}{2(1+\beta)} (k_0 a)^{\beta+1} \, (N_d')^{\beta+1} \tag{16}$$

$$= \frac{(b_d)^{\beta+1}}{2(1+\beta)} (k_0 a)^{\beta+1} N^{\frac{\beta+1}{d}} \,. \tag{17}$$

Here, the dominant terms for $N_d' \gg 1$ are $\propto (N_d')^{\beta+1}$ if $\beta > -1$.

For the case $\beta = 0$, the explicit expression for the integral reads

$$\begin{aligned} J_{cc} & \overset{\beta=0}{\equiv} \frac{k_0 a}{2} \Big[N_d' \left(\mathrm{sinc}(r_- a N_d') + \mathrm{sinc}(r_+ a N_d')\right) \\ & \qquad - \mathrm{sinc}(r_+ a) - \mathrm{sinc}(r_- a) \Big] \qquad (18) \\ & \overset{|r_-|a \ll 1,\; N_d' \gg 1}{\simeq} \frac{k_0 a}{2} N_d' \mathrm{sinc}(r_- a N_d') \\ & \overset{k \to k_0}{\to} \frac{k_0 a}{2} N_d' = \frac{k_0 a}{2} b_d N^{\frac{1}{d}} \,. \end{aligned}$$

In the second step, we focus on wavenumbers k around k_0 (i. e., small $|r_-|a$, for which the terms with r_+ are negligible).

B. $J_{\rm sc}$

Similarly,

$$\begin{aligned} J_{sc} = \frac{\mathrm{i}^{\beta+2}}{4} \cdot \Bigg[& \left(\frac{r_-}{k_0}\right)^{-1-\beta} (\mathrm{sgn}(r_-))^{-2\beta} \\ & \times \Big(- \Gamma(1+\beta, -\mathrm{i}r_- a) \\ & \quad + \Gamma(1+\beta, -\mathrm{i}r_- a N_d') \\ & \quad - (-1)^\beta \Gamma(1+\beta, \mathrm{i}r_- a) \\ & \quad + (-1)^\beta \Gamma(1+\beta, \mathrm{i}r_- a N_d')\Big) \\ + & \left(\frac{r_+}{k_0}\right)^{-1-\beta} \Big(\Gamma(1+\beta, -\mathrm{i}r_+ a) \\ & \quad - \Gamma(1+\beta, -\mathrm{i}r_+ a N_d') \\ & \quad + (-1)^\beta \Gamma(1+\beta, \mathrm{i}r_+ a) \\ & \quad - (-1)^\beta \Gamma(1+\beta, \mathrm{i}r_+ a N_d')\Big)\Bigg] \,. \end{aligned} \tag{19}$$

The asymptotic form for $N'_d \gg 1$ but $r_- \neq 0$ reads

$$J_{sc}(k \neq k_0) \simeq \frac{\mathrm{i}^{\beta+2}}{4} \cdot \Bigg[\left(\frac{r_-}{k_0}\right)^{-1-\beta} (\mathrm{sgn}(r_-))^{-2\beta} \times \Big(- \Gamma(1+\beta, -\mathrm{i}r_- a) - (-1)^\beta \Gamma(1+\beta, \mathrm{i}r_- a) + 2(-1)^\beta (\mathrm{i}r_- a N'_d)^\beta \cos(r_- a N'_d) \Big) + \left(\frac{r_+}{k_0}\right)^{-1-\beta} \Big(\Gamma(1+\beta, -\mathrm{i}r_+ a) - (-1)^\beta \Gamma(1+\beta, \mathrm{i}r_+ a) - 2(-1)^\beta (\mathrm{i}r_+ a N'_d)^\beta \cos(r_+ a N'_d) \Big) \Bigg] . \tag{20}$$

In contrast to the integral J_{cc}, the limit $k \to k_0$ does not yield a scaling $\propto (N'_d)^{\beta+1}$. The case $\beta = 0$ reads

$$J_{sc} \overset{\beta=0}{=} \frac{k_0 a}{2} \Big[\frac{\cos(r_- a N'_d)}{r_- a} - \frac{\cos(r_+ a N'_d)}{r_+ a} - \frac{\cos(r_- a)}{r_- a} + \frac{\cos(r_+ a)}{r_+ a} \Big] \overset{|r_-|a \ll 1}{\simeq} \frac{k_0 a}{2} \cdot \frac{\cos(r_- a N'_d) - 1}{r_- a} . \tag{21}$$

C. J_{cs}

We can rewrite

$$J_{\mathrm{cs}} = \int_{k_0 a}^{k_0 a b_d \sqrt[d]{N}} \mathrm{d}\eta \; \cos(\eta) \sin(k k_0^{-1} \eta) \; \eta^\beta = \left(\frac{k_0}{k}\right)^{\beta+1} \int_{ka}^{kab_d \sqrt[d]{N}} \mathrm{d}\eta \; \sin(\eta) \cos(k_0 k^{-1} \eta) \; \eta^\beta . \tag{22}$$

This is in essence just the integral J_{sc} with k and k_0 interchanged. In particular, for $\beta = 0$, $J_{\mathrm{cs}} = -J_{\mathrm{sc}}$.

D. J_{ss}

For $k \to k_0$, we can write

$$J_{\mathrm{ss}}(k \to k_0) = \int_{k_0 a}^{k_0 a b_d \sqrt[d]{N}} \mathrm{d}\eta \; (1 - \cos^2 \eta) \; \eta^{\beta+1} = \underbrace{(k_0 a b_d \sqrt[d]{N})^{\beta+1}}_{2J_{\mathrm{cc}}(k \to k_0)} - (k_0 a)^{\beta+1} - J_{\mathrm{cc}}(k \to k_0) \overset{N \gg 1}{\simeq} J_{\mathrm{cc}}(k \to k_0) . \tag{23}$$

For $\beta = 0$, the explicit expression reads

$$J_{ss} \overset{\beta=0}{=} \frac{k_0 a}{2} \Big[N'_d \left(\mathrm{sinc}(r_- a N'_d) - \mathrm{sinc}(r_+ a N'_d) \right) + \mathrm{sinc}(r_+ a) - \mathrm{sinc}(r_- a) \Big] \overset{|r_-|a \ll 1,\; N'_d \gg 1}{\simeq} \frac{k_0 a}{2} N'_d \mathrm{sinc}(r_- a N'_d) . \tag{24}$$
